# French Propolis Caffeic Acid Derivatives Protect Skeletal Muscle from Oxidative Damages

**DOI:** 10.3390/biom16040550

**Published:** 2026-04-08

**Authors:** Luis Portillo-Lemus, Barbara Vernus, Béatrice Chabi, Aurélien Lebrun, Guillaume Cazals, Sylvie Rapior, Françoise Fons, Gilles Carnac, Sylvie Morel

**Affiliations:** 1 CEFE, Univ Montpellier, CNRS, EPHE, IRD, 34093 Montpellier, France; 2 DMEM, Univ Montpellier, INRAE, 34000 Montpellier, France; 3 PAC Chimie Balard, Univ Montpellier, CNRS, ENSCM, 34293 Montpellier, France; 4 PhyMedExp, Univ Montpellier, CNRS, INSERM, 34090 Montpellier, France

**Keywords:** propolis functional diversity, oxidative stress, skeletal muscle cells, caffeate derivatives, cytoprotection, lipid peroxidation, food preservation, preventive medicine

## Abstract

Propolis produced by honeybees, *Apis mellifera*, has been valued since ancient times as a remedy for different ailments for its broad medicinal properties. This wide range of biological activities may arise from the production of distinct propolis types within the hive, each serving specific functions and containing unique molecular compositions. In this study, we investigated the effects of four propolis types—masonry, sealing, brood-protection, and intruder-neutralizing—on hydrogen peroxide (H_2_O_2_)-induced oxidative injury in human skeletal muscle cells. Among these, only brood-protection propolis significantly prevented the H_2_O_2_-induced loss of cell viability. Bio-guided fractionation of this active propolis identified five major compounds: benzyl caffeate (BC), caffeic acid phenethyl ester (CAPE), cinnamyl caffeate (CC), prenyl caffeate (PC), and (*E*)-3-methyl-3-butenyl caffeate (MBC), all displaying stronger cytoprotective effects than their ferulate equivalents. We finally demonstrated that propolis extract and its active compounds reduced lipid peroxidation in post-mortem minced mouse skeletal muscle and compared their efficacy to other natural compounds. Chemical analysis of resins from neighboring flora suggested that black poplar (*Populus nigra*) buds are the primary botanical source of these caffeate derivatives. Collectively, these results highlight the functional diversity of hive propolis and its potential applications in food preservation as well as in complementary and preventive medicine.

## 1. Introduction

Propolis is a complex material produced by honeybees (*Apis mellifera*: *Apidae*: *Apinae*) and stingless bees (*Apidae*: *Apinae*: *Meliponini*) through the collection of plant resins and exudates from various botanical sources, including buds, flowers, bark, latex, and wounded tissues [1]. Although considered an animal-derived product, propolis is predominantly of plant origin, as most of its bioactive constituents arise from vegetal resins subsequently mixed by bees with wax, pollen, and salivary enzymes, enhancing its biological properties [2]. Owing to its lipophilic nature and adhesive characteristics, propolis has historically been referred to as “bee glue” and plays a central structural and protective role within the hive [3].

In the hive, propolis fulfills multiple essential functions, including sealing cracks, reinforcing structural integrity, embalming intruders, contributing to thermal insulation, and, most importantly, limiting microbial proliferation in critical areas. This functional diversity is reflected in the etymology of the word propolis, derived from the Greek *pro*- (“in defense”) and *polis* (“city”), meaning “defense of the community.” Although several bee species produce propolis, *Apis mellifera* is recognized as the most efficient producer, yielding up to several hundred grams per hive annually [4].

The remarkable biological activities attributed to propolis are largely explained by its highly complex chemical composition, which comprises several hundred identified compounds. Among these, phenolic compounds, including flavonoids and phenolic acids, are considered major contributors to its biological effects [5]. Among the phenolic constituents of propolis, caffeic acid and its ester derivatives represent a prominent and biologically active class of compounds, particularly in propolis from temperate zones of poplar origin. These caffeates, including caffeic acid phenethyl ester (CAPE) and structurally related esters, have been widely reported to display antioxidant, anti-inflammatory, and cytoprotective properties [6]. Their biological activities are commonly attributed to the presence of the catechol moiety, which confers efficient free radical scavenging capacity and metal-chelating properties, as well as the ability to modulate redox-sensitive signaling pathways. In addition to their direct antioxidant effects, caffeic acid esters have been shown to influence lipid peroxidation, mitochondrial function, and inflammatory responses in various cellular models [6]. However, despite their abundance in propolis and their well-documented bioactivities, the relative contribution of individual caffeate derivatives to the antioxidant and cytoprotective effects of functionally distinct propolis types remains poorly defined, particularly in the context of skeletal muscle oxidative stress [7]. Notably, antioxidant activity has attracted considerable attention due to the central role of oxidative stress in the pathogenesis of numerous chronic diseases [8].

Oxidative stress results from an imbalance between the production of reactive oxygen species (ROS) and antioxidant defenses, leading to oxidative damage of cellular macromolecules such as lipids, proteins, and DNA [9]. In skeletal muscle, excessive ROS production is increasingly recognized as a key contributor to muscle dysfunction, degeneration, and wasting associated with aging and muscle-specific disorders, including muscular dystrophies. Redox homeostasis tightly regulates muscle mass by influencing protein synthesis and degradation pathways, and sustained oxidative stress has been linked to impaired muscle regeneration, mitochondrial dysfunction, altered calcium homeostasis, chronic inflammation, and activation of catabolic signaling pathways such as NF-κB [10,11].

Despite growing interest in antioxidant supplementation as a strategy to preserve muscle function, clinical evidence supporting their efficacy remains limited and inconsistent [12,13,14,15]. One major limitation lies in the empirical selection of antioxidant compounds, often based on availability rather than biological effectiveness. Moreover, several antioxidant molecules have been reported to exert deleterious effects on muscle cell differentiation and tissue homeostasis, highlighting the need for rigorous preclinical evaluation of both efficacy and toxicity in relevant cellular models [16]. In addition, food processing industry is always in need of new natural compounds to preserve food from natural oxidation such as lipid peroxidation in processed meat which can be evaluated with the commonly used TBARS (Thiobarbituric acid reactive substances) assay. In this context, natural products with complex and synergistic antioxidant profiles, such as propolis, represent promising candidates. However, most studies consider propolis as a homogeneous product, despite increasing evidence that distinct propolis types coexist within the hive and may serve specialized biological functions, potentially associated with specific chemical signatures [17].

In the present study, we used a hydrogen peroxide (H_2_O_2_)-induced oxidative stress assay in human skeletal muscle cells to evaluate the antioxidant and cytoprotective effects of four functional types of French propolis. We developed a bioassay-guided fractionation approach to identify the specific biomolecules responsible for the observed cytoprotective effects and to assess their ability to prevent oxidative damage in cellular and ex vivo skeletal muscle models. This work aims to highlight the functional and chemical specialization of beehive propolis and to emphasize its potential applications in muscle protection, food preservation, and preventive medicine.

## 2. Materials and Methods

### 2.1. Propolis Material

The propolis samples used for bioassay-guided fractionation were collected in 2023 from 10 beehives located in Saint-Gal-sur-Sioule, Puy-de-Dôme, France (46.104244° N, 3.013440° E). Prop-A (brood-protection propolis) was collected in spring 2023 (April–May), corresponding to the peak queen egg-laying period. The colonies consisted of Buckfast honeybees kept in Dadant hives (10 brood frames in the brood chamber and 9 frames in the supers). Samples were obtained using propolis traps designed to remove propolis pellets from the hind legs of worker bees upon hive entry (See Figure S1, Supplementary Information). Prop-B (sealing propolis) was collected throughout the year by scraping propolis deposited between hive boxes and at the edges of the frames. Prop-C (intruder-neutralizing propolis) was collected in autumn, a period during which other insects, such as moths, seek shelter and may become trapped inside the hive. Prop-D (masonry propolis) was collected year-round by scraping the propolis bridges constructed by bees between the queen excluder and the main frames.

The propolis samples used to evaluate the presence of caffeates and test the activity of these propolis are commercial samples of the Propolia brand from Brazil, France and the European Union; the bee subspecies, hive type, apiary location, and the exact date of propolis collection were unknown. Other propolis samples were collected in 2025 from different regions of Southern of France, in Puechabon (Hérault, 43.716199° N, 3.600176° E) and in Aubignosc (Alpes-de-Haute-Provence, 44.135001° N, 5.939326° E) from Miellerie Maccario, the colonies consisted of Buckfast honeybees kept in Dadant hives, and the propolis samples were collected in spring 2025 (April–May). All samples were stored at room temperature in airtight containers and protected from light.

### 2.2. Plant Material

The resin from *Aesculus hippocastanum*, *Cedrus atlantica*, *Pinus halepensis* and *Populus nigra* were collected in spring 2023 (April–May) near the apiary located in Saint-Gal-sur-Sioule (Puy-de-Dôme, France). In addition, resins from other black poplar (*Populus nigra*) trees were collected in southern France in spring 2025, to assess potential chemical variations in resin composition associated with geographical origin.

### 2.3. Extraction Processes

Propolis samples (10 g, Prop-A, -B, -C, -D) were extracted with absolute ethanol using ultrasound-assisted extraction. Briefly, propolis was mixed with ethanol (1:20, *w*/*v*) and heated at 40 °C under ultrasonic treatment for 15 min. The mixture was then filtered, and the solvent was evaporated to dryness under reduced pressure using a rotary evaporator. For bioassay-guided fractionation, the same extraction procedure was applied at a larger scale (100 g of propolis extracted with 2 L of absolute ethanol).

### 2.4. Bioassay-Guided Fractionation of Caffeate Derivatives

To identify the compound(s) responsible for the antioxidant activity of propolis, we used a bioassay-guided fractionation method. At each step of the purification, we evaluated the ability of the obtained fractions to protect myoblasts against H_2_O_2_-induced cell death (assay described in Section 2.9). The propolis extract (Prop-A,100 g) was subjected to successive liquid–liquid partitioning using immiscible solvent systems of increasing polarity. The extract was first partitioned between cyclohexane (1 L) and water (1 L), followed by partitioning between water and dichloromethane (1 L), and finally between water and ethyl acetate (1 L). Dichloromethane fraction (Prop-A-2, 10 g) was submitted to permeation gel chromatography on a Sephadex LH-20 column (2.3 × 60 cm, 70 g LH-20). The elution was performed from 100% EtOH (3 L). The elution was finalized with 100% acetone (50 mL). After thin-layer chromatography (TLC) analysis of all the 20 mL tubes collected, seven fractions were obtained (1 to 7).

We decided to follow the bioactivity-guided purification with fraction 4 (Prop-A-2-4, 700 mg). Fraction 4 solubilized in EtOH was submitted to permeation gel chromatography on a Sephadex LH-20 column (1.5 × 40 cm, 20 g LH-20). The elution was performed from 100% EtOH (1 L). The elution was finalized with 100% acetone (50 mL). After thin-layer chromatography (TLC) analysis of all the 20 mL tubes collected, eight fractions were obtained (Prop-A-2-4-1 to 8).

The 2 (50 mg) and 3 (35 mg) fractions (Prop-A-2-4-2+3) were combined and further purified by preparative high-performance thin-layer chromatography (HPTLC) using silica gel 60 F254 MS-grade plates (Merck KGaA, Darmstadt, Germany). Samples were applied as bands onto the plates and developed in a cyclohexane/ethyl acetate solvent system (40:60, *v*/*v*). After migration, compounds were visualized under UV light (254 and 365 nm), and the bands of interest were scraped from the plate, eluted from the silica with ethyl acetate solvent, filtered, and the solvent was removed under reduced pressure using a rotary evaporator; 51 mg were obtained for subsequent analyses.

### 2.5. Mass Chromatography (MS) Analyses

Compounds contained in Prop-A-2-4-2+3-b fraction were characterized by LC-MS using a Synapt G2-S high-definition mass spectrometry system (Waters Corp., Milford, MA, USA) equipped with an electrospray ionization source to characterize the elemental composition of parent and fragment ions according to the procedure previously described [18].

### 2.6. Nuclear Magnetic Resonance Spectroscopy (NMR) Analyses

NMR spectra from active fraction and caffeate and ferulate derivatives were recorded at 298 K on a Bruker Avance NEO 600 MHz NMR spectrometer using Bruker TCI Prodigy^®^ Nitrogen Cryoprobe (Fällanden, Switzerland). ^13^C UDEFT sequence was used with spectral width of 36,000 Hz and 512 scans. 2D Homonuclear spectra ^1^H-^1^H g-COSY was recorded using data matrice of 256 real (t1) × 2048 (t2) complex data points; 8 scans per t1 increment with spectral width of 7200 Hz in both dimensions were used. In addition, 2D heteronuclear spectra ^13^C-^1^H g-edited HSQC and ^13^C-^1^H g-HMBC (8–16 scans, 256 real (t1) × 2048 (t2) complex data points) were acquired to assign compounds. Chemical shifts δ are given in ppm and coupling constants J are provided in Hz. NMR spectra were calibrated with residual protic solvent peak (e.g., CDCl_3_: ^1^H: 7.26 ppm, ^13^C: 77.16 ppm). Multiplicities are defined as follows: s = singlet, d = doublet, t = triplet, br = broad; and coupling constants are provided in Hz. Spectra were processed and visualized with Topspin 4.5.0, Bruker Biospin (Fällanden, Switzerland). CDCl_3_ was purchased from Innovachem, France.

### 2.7. HPLC Analysis

Chromatographic separation and detection were performed on an Ultimate 3000 (Thermo Fisher Scientific Inc., San Jose, CA, USA) instrument that included a quaternary pump, a degasser, an automatic sampler and a UV Diode Array Detector. The system was operated using Chromleon software, version 7.0. Chromatographic separation was achieved on an ODS Hypersyl C18 column (250 mm, 4.6 mm, 5 µm, Thermo Fisher Scientific Inc., San Jose, CA, USA), with a column temperature maintained at 35 °C.

Samples were eluted at a flow rate of 1.2 mL/min, using solvent A (water/formic acid 99.9:0.1 *v*/*v*) and solvent B (acetonitrile). The gradient used for the analysis of samples was expressed as a percentage of B, where 0–2 min: 5%, 10 min: 26%, 120 min: 26%, 170 min: 36%, 195 min: 70%, 197 min: 100%, 205 min: 100%, 210 min: 5%; 212 min: 5%. The UV/vis spectra were recorded in the 200–400 nm range and chromatograms were acquired at 254, 280 and 330 nm. Extracts were analyzed at 5 mg/mL.

### 2.8. Primary Cultures of Human Myoblasts

We produce our primary culture of human myoblasts: Quadriceps muscle biopsies were obtained from one 19-year-old male (M19; Healthy male), at the Centre Hospitalier Universitaire Lapeyronie (Montpellier, France). Donor signed an informed written consent after the description of the protocol (Authorization N° DC-2008-594). Briefly, myoblasts (muscle progenitor cells) were purified from the muscle biopsies using an immunomagnetic sorting system with magnetic activated cell sorter (MACS) microbeads linked to an antibody against N-CAM (CD56) (Ref: 130-050-401; Miltenyi Biotec). After purification, H19 cells were at least 96% positive for desmin (a muscle marker) by immunofluorescence. Myoblasts were cultured on collagen-coated dishes (ref: 356456, Corning, NY, USA) in DMEM/F12 medium with 10% fetal bovine serum (FBS), 0.1% Ultroser G and 1 ng/mL of human basic fibroblast growth factor (proliferation medium), as previously described [19,20,21].

Primary human myoblasts retain the intrinsic heterogeneity and physiological characteristics of the tissue of origin, whereas immortalized cell lines frequently undergo genetic drift and phenotypic adaptation during long-term culture [22,23,24,25,26,27]. Therefore, primary cultures provide a biologically relevant model that better reflects native human muscle biology.

### 2.9. Cell Death Quantification

Myoblasts were seeded at 1.10^5^ in 35 mm collagen-coated dishes, cultured in proliferation medium for 24 h, pre-incubated or not with the tested compounds for 24 h and then incubated or not with a lethal concentration of hydrogen peroxide (H_2_O_2_), a strong pro-oxidant/pro-apoptotic compound, for 24 h. The optimal H_2_O_2_ concentration was the concentration required to kill between 30 and 50% of total cells and was established before each experiment. Total cells (non-adherent cells and adherent cells) were labeled with the Muse^®^ Count and Viability Kit (ref: MCH100102, Luminex, Austin, TX, USA) to detect apoptotic cells followed by analysis with a Fluorescence Activated Cell Sorting (FACS) Muse apparatus (Millipore, Molsheim, France).

### 2.10. Lipid Peroxidation Analysis

#### 2.10.1. Post-Mortem Mouse Muscle Sampling and Preparation

The experimental protocol was in strict accordance with the European directives (86/609/CEE) and was approved by the Ethical Committee of the Occitanie Region.

#### 2.10.2. Gastrocnemius and Quadriceps

Muscles from six-month-old C57BL/6 male mice were removed and immediately placed on ice. Skeletal muscles were then minced with sterile scissors for 5 min and divided in 150 mg batches for each molecule and 300 mg for the control batch.

#### 2.10.3. Evaluation of the Antioxidant Potential of Propolis Extracts

To evaluate the antioxidant properties of natural product, batches of minced muscle were mixed with propolis or some of its compounds obtained by bio-guided fractionation.

Prop-A, an extract from propolis, and five of its bioactive compounds were analysed: Caffeic acid phenethyl ester (CAPE), 3-Methyl-3-butenyl-(*E*)-caffeate (MBC), Benzyl caffeate (BZC), Cinnamyl caffeate (CC) and Prenyl caffeate (PC). Positive control batches were treated with butylated hydroxytoluene (BHT, B1378, Sigma Aldrich/Merck, Saint-Quentin-Fallavier, France), carnosid acid (CA, 0.01%; 15 μL/150 mg) and E392, an additive used in food industry better known as rosemary extract. A negative control batch was mixed only with ethanol (CTRL, 0.01%; 30 μL/300 mg). This batch was divided in two portions (150 mg) using a weighing cup. All tested molecules were dissolved in ethanol (0.01%, *w*/*w* minced muscle, 15 μL/150 mg), the concentration of the stock solution was corrected according to the molecular weight of the molecule. Carnosic acid 0.01% was used as reference.

All portions were packaged in a same polypropylene box with damp paper and covered with a film to maintain a humid atmosphere. The portions were stored at 4 ± 1 °C in the dark for 7 days. One portion of control batch (Day 0) was immediately homogenized in 50 mM phosphate buffer (pH 7.0) (1:9 *w*/*v*) with an Ultra-Turrax homogenizer (30 s, 16,000 rpm). The fraction of homogenate needed for thiobarbituric acid reactive substances (TBARS) measurement was quickly frozen. The same procedure was adopted for a 7-day time point.

#### 2.10.4. TBARS Measurement in Post-Mortem Mouse Muscle

The lipid peroxidation index was determined in muscle homogenates by measuring TBARS as previously described in [21,28].

Results were expressed as nanomoles of TBARS per gram of fresh tissue and presented as mean ± standard deviation of three independent experiments.

### 2.11. Statistical Analysis

All experiments were conducted with three independent biological replicates per condition. For each replicate, the measured response (e.g., percentage of cell death or metabolite content) was calculated and used for statistical analysis. Data are presented as mean ± standard deviation (SD), reflecting the variability between biological replicates.

Statistical differences among conditions were evaluated using one-way analysis of variance (ANOVA), followed by Tukey’s honestly significant difference (HSD) *post hoc* test for multiple comparisons. Differences were considered statistically significant at *p* < 0.05. Statistical analyses and graphical representations were performed using R software (R version 4.3.3 (29 February 2024 ucrt)—“Angel Food Cake” Copyright (C) 2024 The R Foundation for Statistical Computing Platform: x86_64-w64-mingw32/x64 (64-bit)) and the ggplot2 package (version 3.5.1, available at https://cran.r-project.org/package=ggplot2, accessed on 24 March 2026).

## 3. Results

### 3.1. Cytoprotective Activity of Different Types of Propolis and Bioactivity-Guided Separation of Antioxidant Compounds

**Figure 1:**
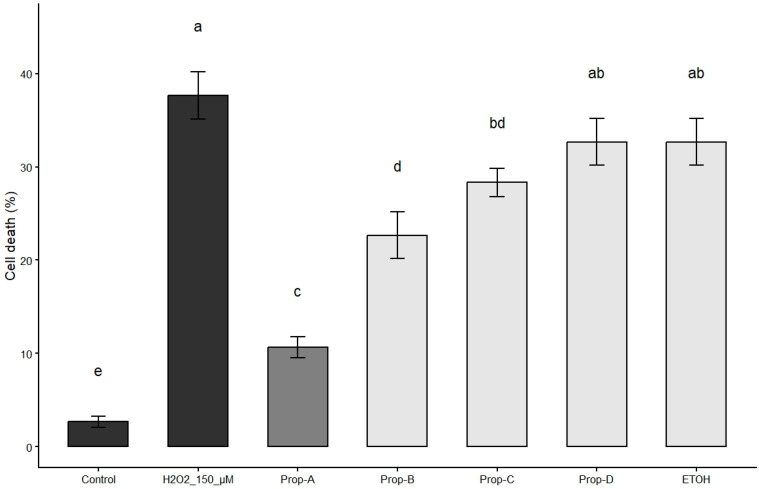
Propolis type selection. Cytoprotective activities of ethanolic extracts from four distinct functional types of propolis: Prop-A, Brood-protection propolis; Prop-B, Sealing propolis; Prop-C, Intruder-neutralizing propolis; Prop-D, Masonry propolis; ETOH, absolute ethanol. Cell death quantification (percentage of all cells) in human myoblasts incubated with propolis ethanolic extracts at 5 μg/mL prior to incubation with H_2_O_2_ to evaluate cytoprotective activity. Data are shown as mean ± SD (*n* = 3). Different letters indicate significant differences (one-way ANOVA followed by Tukey’s HSD test, *p* < 0.05).

We previously demonstrated that exposure of primary human myoblasts (skeletal muscle precursor cells) to H_2_O_2_ induces a marked increase in apoptosis in adherent cultures [9]. This cellular model was used to evaluate the cytoprotective activities of ethanolic extracts from four distinct functional types of propolis. A pre-treatment with propolis at 5 µg/mL for 24 h significantly reduced H_2_O_2_-induced cell death (Figure 1). Notably, among the four types of propolis tested, only brood-protection propolis significantly reduced cell death percentage after H_2_O_2_ exposure and was therefore selected (Prop-A) for subsequent bioassay-guided fractionation to identify the molecules responsible for this activity.

**Figure 2:**
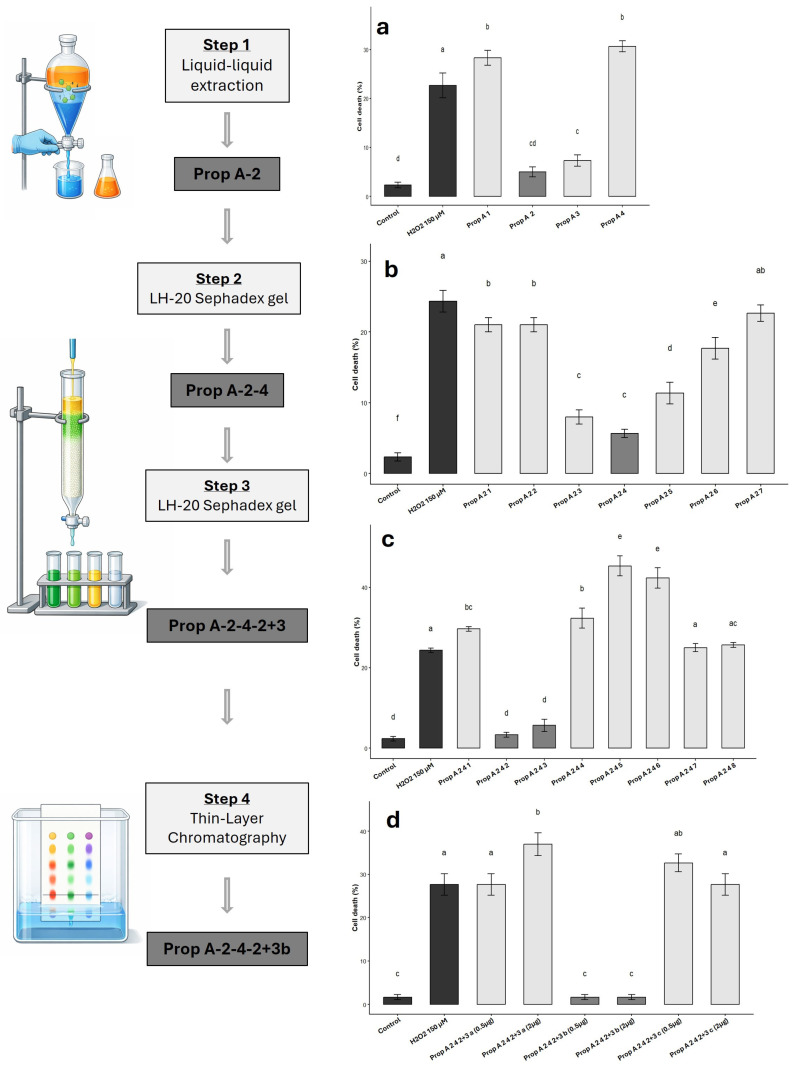
Bioactivity-guided separation of cytoprotective compounds from brood-protection propolis. Schematic representation of the successive separation and purification steps (Steps 1–4) and of the fractions selected at each stage (**left**). Cytoprotective activity of the selected fractions at each purification (**a**–**d**) (**right**). Cell death quantification (percentage of total cells) was measured in human myoblasts pre-incubated with the indicated fractions ((**a**–**c**) 5 µg/mL and (**d**) 2 and 0.5 µg/mL) prior to exposure to H_2_O_2_-induced oxidative stress. Data are shown as mean ± SD (*n* = 3). Different letters indicate significant differences (one-way ANOVA followed by Tukey’s HSD test, *p* < 0.05).

Bioactivity-guided separation of cytoprotective compounds on brood-protection propolis is based on four steps as follows: To identify the molecules responsible for the cytoprotective activity, brood-protection propolis was subjected to liquid–liquid extraction using four solvents of increasing polarity: cyclohexane, dichloromethane, ethyl acetate, and water (Figure 2, Step 1). Each resulting fraction was evaluated for its cytoprotective activity using the same human skeletal muscle cell model (Figure 2a). Among the four fractions tested, only the dichloromethane and ethyl acetate extracts exhibited significant protective activity against H_2_O_2_-induced oxidative stress. Despite similar cytoprotective activities observed for the Prop-A-2 dichloromethane and Prop-A-3 ethyl acetate fractions (Figure 2a), TLC analysis (UV 254 and 366 nm; H_2_SO_4_ 0.5 M in methanol) showed a substantially simpler chromatographic profile for Prop-A-2, displaying three spots versus approximately fifteen for Prop-A-3, indicating a lower chemical complexity and easier purification. Based on these results, Prop-A-2 dichloromethane extract was selected for subsequent analyses.

During the second purification step, the Prop-A-2 extract was fractionated by liquid chromatography on Sephadex LH-20 with absolute ethanol as the eluent, resulting in seven fractions. The Prop-A-2-4 fraction was chosen for subsequent experiments based on a combination of cytoprotective efficacy, reduced chemical complexity observed by TLC analysis, and sufficient fraction mass for further purification (Figure 2b, step 2).

Following a second chromatographic separation on Sephadex LH-20 using absolute ethanol as the eluent, eight subfractions were obtained from the Prop-A-2-4 fraction (Figure 2, Step 3). The Prop-A-2-4-2 and Prop-A-2-4-3 subfractions displayed comparable cytoprotective effects (Figure 2c). Despite the lower complexity observed for Prop-A-2-4-2 by TLC analysis, both subfractions were selected to ensure adequate material for subsequent purification.

The pooled Prop-A-2-4-2 and Prop-A-2-4-3 fractions were further separated by preparative thin-layer chromatography. Three distinct bands (a, b, and c) were detected under UV light at 254 nm, isolated, and subsequently evaluated for cytoprotective activity. Among them, only the Prop-A-2-4-2+3-b band displayed cytoprotective activity comparable to that of the control (Figure 2d).

**Figure 3:**
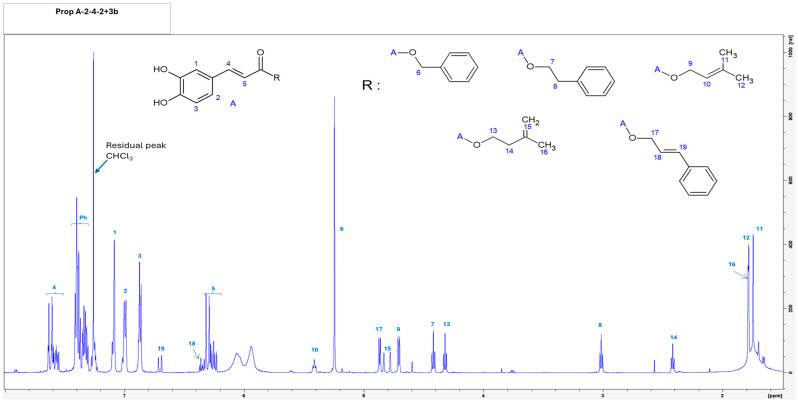
Structural elucidation of cytoprotective biomolecules isolated from brood-protection propolis. Structural characterization was performed using nuclear magnetic resonance (NMR) spectroscopy and mass spectrometry analyses, leading to the identification of 1, 2, 3, 4, 5 caffeic acid core; 6 benzyl caffeate (BC); 7, 8 caffeic acid phenethyl ester (CAPE); 9, 10, 11, 12 (*E*)-3-methyl-3-butenyl caffeate (MBC); 13, 14, 15, 16 prenyl caffeate (PC); and 17, 18, 19 cinnamyl caffeate (CC).

The bioactive Prop-A-2-4-2+3-b fraction containing a mixture of five cytoprotective compounds was obtained. Structural elucidation was achieved by nuclear magnetic resonance (NMR) spectroscopy (Figure 3) combined with mass spectrometry analysis (Figures S2 and S3, Supplementary Data). These analyses led to the identification of benzyl caffeate (BC), caffeic acid phenethyl ester (CAPE), cinnamyl caffeate (CC), prenyl caffeate (PC), and (*E*)-3-methyl-3-butenyl caffeate (MBC).

**Figure 4:**
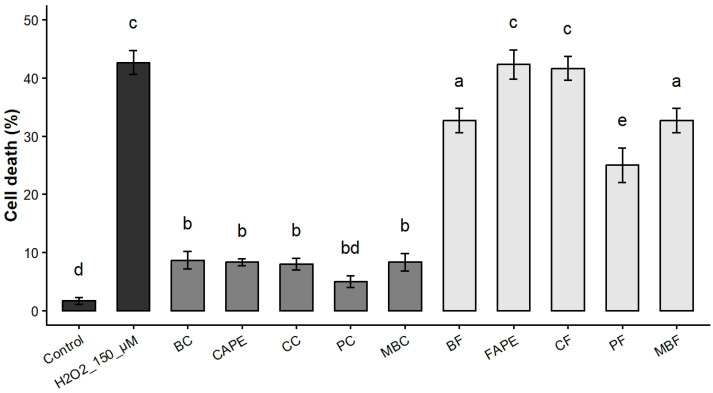
Cytoprotective activities of five caffeates and five ferulates standards. Cell death quantification (percentage of all cells) in human myoblasts incubated with all commercial standards at 2 μM prior to incubation with H_2_O_2_. Data are shown as mean ± SD (*n* = 3). Different letters indicate significant differences (one-way ANOVA followed by Tukey’s HSD test, *p* < 0.05). Benzyl caffeate (BC), caffeic acid phenethyl ester (CAPE), 3-cinnamyl caffeate (CC), prenyl caffeate (PC), and (*E*)-3-methyl-3-butenyl caffeate (MBC), benzyl ferulate (BF), ferulic acid phenethyl ester (FAPE), cinnamyl ferulate (CF), prenyl ferulate (PF), and (*E*)-3-methyl-3-butenyl ferulate (MBF).

To determine whether the cytoprotective activity was attributable to a single compound, namely the most abundant species in the fraction, each caffeate from commercial source was individually tested. In parallel, the corresponding commercial ferulate analogues were also evaluated, differing solely by the substitution of a hydroxyl group with a methoxy group at the 5-position of the aromatic ring. Functional analyses revealed that cytoprotective activity was exclusively associated with the caffeic acid esters, whereas none of the ferulate derivatives exhibited protective effects (Figure 4). Moreover, all caffeate compounds displayed comparable antioxidant activity, whether tested individually (Figure 4) or as a mixture (Figure 2d). Notably, no increase in cell death was observed at concentrations up to 100-fold higher than those required to elicit cytoprotective effects, indicating low cytotoxicity of these compounds (Figure S4, Supplementary Data).

### 3.2. Comparative Analysis of Caffeate Composition and Cytoprotective Activity in Propolis and Plant Resins

**Figure 5:**
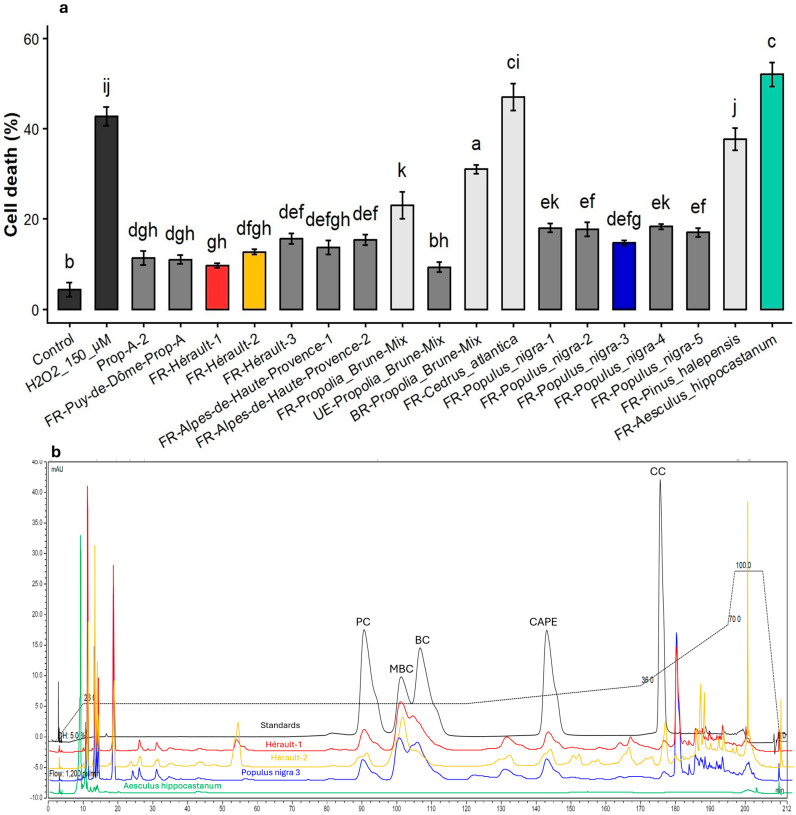
Cytoprotective activities of eight propolis samples from different geographical origins and of resin extracts from four tree species located in the vicinity of the apiary that produced Prop-A. (**a**) Cell death quantification (percentage of all cells) in human myoblasts incubated with all commercial standards at 5 µg/mL prior to incubation with H_2_O_2_. Data are shown as mean ± SD (*n* = 3). Different letters indicate significant differences (one-way ANOVA followed by Tukey’s HSD test, *p* < 0.05) (**b**) HPLC chromatogram at 320 nm of five standards of caffeates (Black color) Benzyl caffeate (BC), caffeic acid phenethyl ester (CAPE), 3-cinnamyl caffeate (CC), prenyl caffeate (PC), and (*E*)-3-methyl-3-butenyl caffeate (MBC) and some samples of propolis: Hérault-1 (red) and Hérault-2 (yellow), *Populus nigra* 3 associated with good activity (blue) and *Aesculus hippocastanum* with no activity (no caffeate) (green) to illustrate the chemical composition of each of the resins. The dashed line represents the dilution gradient.

To further support these findings, the cytoprotective potential of eight propolis samples from different geographical origins was evaluated, using Prop-A and Prop-A-2 as controls (Figure 5a). Cytoprotective activity was observed exclusively in propolis samples containing at least one of the five caffeic acid esters at concentrations above the HPLC detection limit. In parallel, the presence of these caffeate derivatives was investigated in resin extracts from four tree species located in the vicinity of the apiary that produced the propolis used for bioassay-guided fractionation. Among the plant species analyzed, only black poplar (*Populus nigra*) resin contained all five caffeic acid esters identified in brood-protective propolis. Consistently, cytoprotective activity was detected exclusively in *P. nigra* resin extracts (Figure 5b).

### 3.3. Lipid Peroxidation

To extend the role of Prop-A and its five caffeic acid esters (CAPE, MBC, BC, CC, PC) to food preservation, we evaluated their protective effect on lipid peroxidation in *post-mortem* minced skeletal muscle. This evaluation was conducted in comparison with both commonly used synthetic and natural antioxidants such as butylated hydroxytoluene (BHT), carnosic acid (CA) and E392, a food additive better known as rosemary extract containing carnosic acid.

Indeed, lipid peroxidation, originating from free radicals induced oxidation of biological membranes, appears over time in processed meat. This can be delayed by the addition of antioxidants. In laboratory, such a process can be reproduced using minced muscle of mice refrigerated at 4 °C for 7 days and measured using the TBARS assay [17].

**Figure 6:**
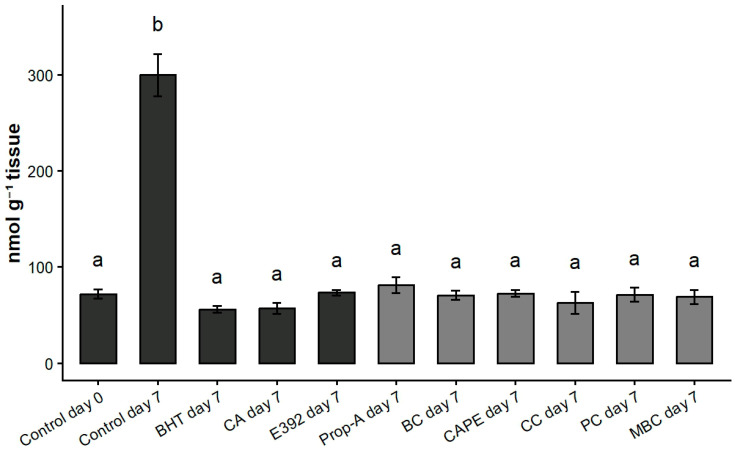
Protective effect of propolis extract and its caffeic derivatives esters on lipid peroxidation in minced mouse skeletal muscle. Minced mice muscles were mixed with ethanol (CTRL), butylated hydroxytoluene (BHT), carnosic acid (CA), E392, Propolis extract (Prop-A), Benzyl caffeate (BC), Caffeic acid phenethyl ester (CAPE), 3-Cinnamyl caffeate (CC), Prenyl caffeate (PC) and (*E*)-3-methyl-3-butenyl caffeate (MBC), dissolved in ethanol. At day 0 and day 7 of refrigerated storage (4 °C), lipid oxidation was evaluated by TBARS quantification; Data are shown as mean ± SD (*n* = 3). Different letters indicate significant differences (one-way ANOVA followed by Tukey’s HSD test, *p* < 0.05).

As expected, lipid oxidation, quantified by TBARS analysis, increased significantly in untreated minced muscle (Control) after 7 days at 4 °C and decreased following treatment with BHT, CA and E392 (Figure 6). Prop-A and its five compounds reduced lipid peroxidation to the same level as three reference antioxidants in minced muscle, compared to the control group, after the 7th day of storage (Figure 6).

## 4. Discussion

In this study, using a bioassay-guided fractionation strategy, we identified five caffeic acid esters, benzyl caffeate, caffeic acid phenethyl ester, cinnamyl caffeate, prenyl caffeate, and (*E*)-3-methyl-3-butenyl caffeate as the main antioxidant compounds responsible for the cytoprotective activity of brood-protection propolis against oxidative stress-induced cell death in human skeletal muscle cells.

Propolis is a well-established source of natural antioxidants and its broad spectrum of biological activities has been recognized since antiquity [29,30]. Analyses of propolis samples from diverse geographical origins reveal substantial variability in their chemical composition, which strongly influences their biological properties [31,32,33,34]. Despite this compositional diversity, propolis consistently exhibits pronounced antibacterial, antiatherogenic, antifungal, antioxidant, and antiviral activities, as well as antiproliferative and proapoptotic effects [35]. Certain propolis types have been reported to display enhanced anti-inflammatory, regenerative, estrogenic, and anesthetic properties, along with selective proapoptotic activity against cancer cells [36,37]. In addition, cardioprotective and hepatoprotective effects of propolis have been documented [29].

Although propolis is widely recognized as a rich source of flavonoids and phenolic acids, our results reveal that, in brood-protection propolis, caffeic acid esters, rather than flavonoids, are the primary contributors to cytoprotective activity [38,39]. The exclusive detection of these five caffeates in *Populus nigra* resin and their strict association with cytoprotective activity across multiple propolis samples strongly support a functional and botanical specialization of this propolis type [6,40].

The comparison between caffeic acid esters and their corresponding ferulate analogues highlights the critical role of the catechol moiety in antioxidant activity. Substitution of the hydroxyl group by a methoxy group on the aromatic ring completely abolished the cytoprotective effects, emphasizing the importance of hydroxyl groups for radical scavenging and redox modulation. Notably, all caffeate derivatives exhibited comparable activity, whether tested individually or as a mixture, suggesting a functional redundancy within this chemical class.

Oxidative stress plays a central role in skeletal muscle degeneration, aging, and impaired regeneration. Myoblasts are particularly sensitive to oxidative insults due to elevated basal ROS levels and limited antioxidant defenses [28,41,42]. Previous studies have shown that reducing oxidative stress improves myoblast survival and enhances the efficacy of muscle cell transplantation. In this context, the ability of caffeic acid esters to protect human myoblasts from oxidative damage highlights their potential relevance for strategies aimed at improving muscle regeneration and cell-based therapies [43].

Lipid peroxidation represents a major consequence of oxidative stress, leading to membrane destabilization, altered cellular signaling, and irreversible tissue damage. In skeletal muscle, lipid peroxidation contributes to impaired membrane integrity, mitochondrial dysfunction, and loss of contractile function. In the present study, both brood-protection propolis extract and its purified caffeic acid esters markedly reduced lipid peroxidation in *post-mortem* minced skeletal muscle, highlighting their capacity to protect complex biological matrices beyond isolated cellular systems. The efficiency of these compounds in limiting lipid oxidation is consistent with the chemical features of caffeic acid esters, whose lipophilic side chains facilitate membrane association while the catechol moiety enables efficient scavenging of lipid-derived radicals. This dual property likely underlies their superior performance compared to more hydrophilic antioxidants and supports their relevance in contexts where lipid oxidation is a primary driver of quality loss, such as muscle degeneration and food spoilage [29].

Extensive research has documented the antioxidant properties of propolis in a wide range of biological systems. Numerous studies have reported protective effects on macrophages, hepatocytes, erythrocytes, and cancer cells [44,45,46,47,48,49,50,51,52], primarily attributed to its high content of phenolic compounds, flavonoids, and aromatic acids [1,29,30]. More generally, these compounds are known to modulate oxidative stress and inflammation pathways, which play a central role in the pathophysiology of chronic diseases [8]. However, most of these studies rely on crude extracts, which prevents the identification of the specific molecules responsible for the observed bioactivities. This limitation is further compounded by the marked chemical variability of propolis depending on its botanical and geographical origin [33,34].

Our work extends these observations by characterizing, at the molecular level, the cytoprotective activity of a functionally specialized propolis type in a skeletal muscle model. This is particularly relevant given the high susceptibility of skeletal muscle to oxidative stress under conditions such as aging, intense exercise, or pharmacological stress [12,16]. Although previous studies suggest that propolis may enhance antioxidant defenses and muscle function [7,13,14], the mechanisms involved at the cellular level remain poorly described. In contrast, bio-guided approaches applied to plant-derived compounds have demonstrated their effectiveness in linking specific molecules to functional effects in muscle cells [19,21,28].

A similar gap exists in studies focusing on the chemical characterization of propolis. Numerous reports provide detailed inventories of compounds from propolis samples from different regions, highlighting its significant chemical diversity [3,4,31,32]. For example, ten major constituents were identified in Chinese propolis derived from *Populus canadensis* [6], including caffeic acid esters that were also detected in the present study. Similarly, recent analyses of Mexican and Egyptian propolis have confirmed the presence of bioactive phenolics associated with antioxidant and antimicrobial properties [2,3]. However, without functional validation, these compounds remain putative contributors, and their respective roles in the biological activity of propolis cannot be established.

Most studies have predominantly focused on the geographical and botanical origin of propolis, paying relatively little attention to its functional heterogeneity within the hive itself [32,34,40]. Propolis is not a uniform material; it is used by bees in different structural and biological contexts, which may influence both its composition and function [53]. In this regard, our findings highlight that the most biologically active propolis fraction corresponds to a thin layer deposited by bees immediately after worker emergence and prior to new oviposition. This specific temporal and spatial use within the hive suggests a specialized biological role, potentially linked to brood cell sanitation or protection against oxidative and microbial stress. Such observations emphasize that, beyond geographic variability, the functional context of propolis deposition within the hive may represent a critical and largely overlooked determinant of its chemical composition and bioactivity.

## 5. Conclusions

This study demonstrates that brood-protection propolis represents a functionally specialized hive product whose antioxidant activity is primarily driven by a defined set of caffeic acid esters originating from *Populus nigra*. By combining bioassay-guided fractionation with relevant cellular and ex vivo protective assays, we provide a mechanistic insight into the structure–activity relationships underlying the cytoprotective effects of these compounds. Beyond their relevance to skeletal muscle biology, these findings also support potential applications of caffeic acid esters in food preservation and preventive nutrition, where oxidative stability is a major concern. Future studies will be required to further investigate their bioavailability, metabolism, and efficacy in vivo.

## Data Availability

The original contributions presented in this study are included in the article/Supplementary Material. Further inquiries can be directed to the corresponding authors.

## Supplementary Materials

The following supporting information can be downloaded at: https://www.mdpi.com/article/10.3390/biom16040550/s1, Figure S1: Propolis traps designed to remove propolis pellets from the hind legs of worker bees upon hive entry. Figure S2: LC-MS Analysis of Prop-A-2-4-2+3b fraction. Benzyl caffeate (BC), prenyl caffeate (PC), and (*E*)-3-methyl-3-butenyl caffeate (MBC). Figure S3: LC-MS Analysis of Prop-A-2-4-2+3b fraction. Caffeic acid phenethyl ester (CAPE), 3-cinnamyl caffeate (CC). Figure S4: Cytotoxicity of five caffeates standards.
