# Supplementary material for: French Propolis Caffeic Acid Derivatives Protect Skeletal Muscle from Oxidative Damages
Source: Biomolecules. 2026 Apr 8;16(4):550. doi: 10.3390/biom16040550 (PMC13113133; doi:10.3390/biom16040550)
Supplement: Supplementary file 1 [file biomolecules-16-00550-s001.zip › biomolecules-4206801-supplementary.pdf]

**Supplementary Materials:**

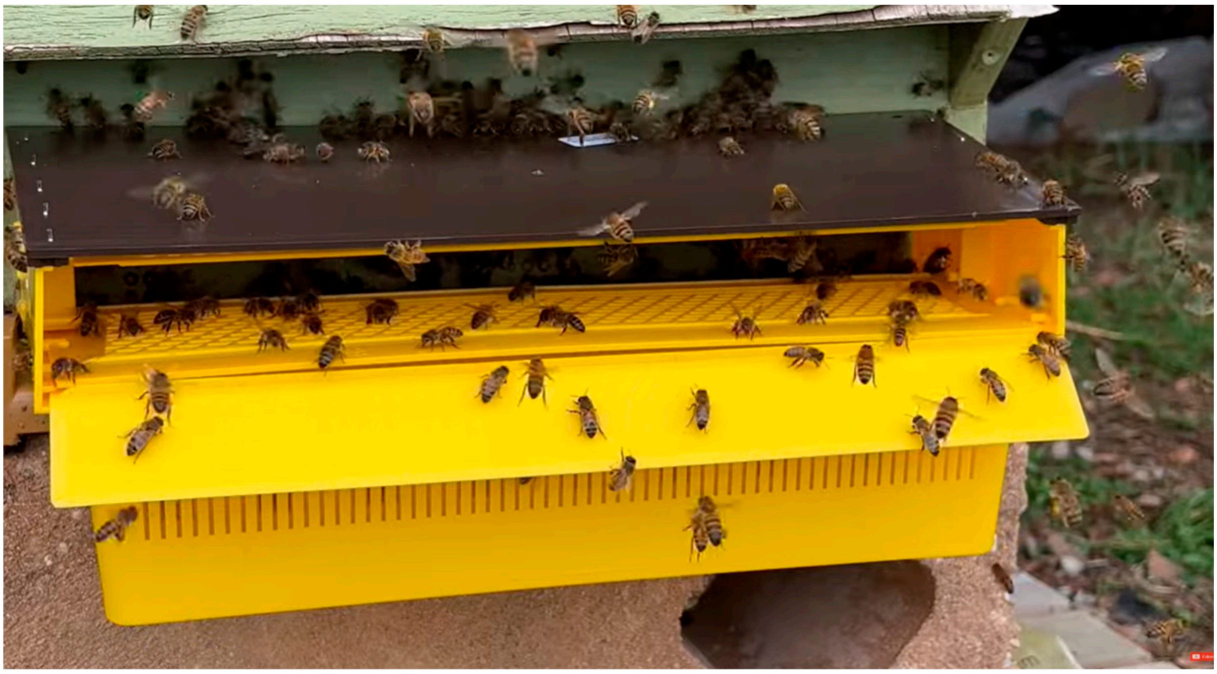

**Figure S1: Propolis traps designed to remove propolis pellets from the hind legs of worker bees upon hive entry.**

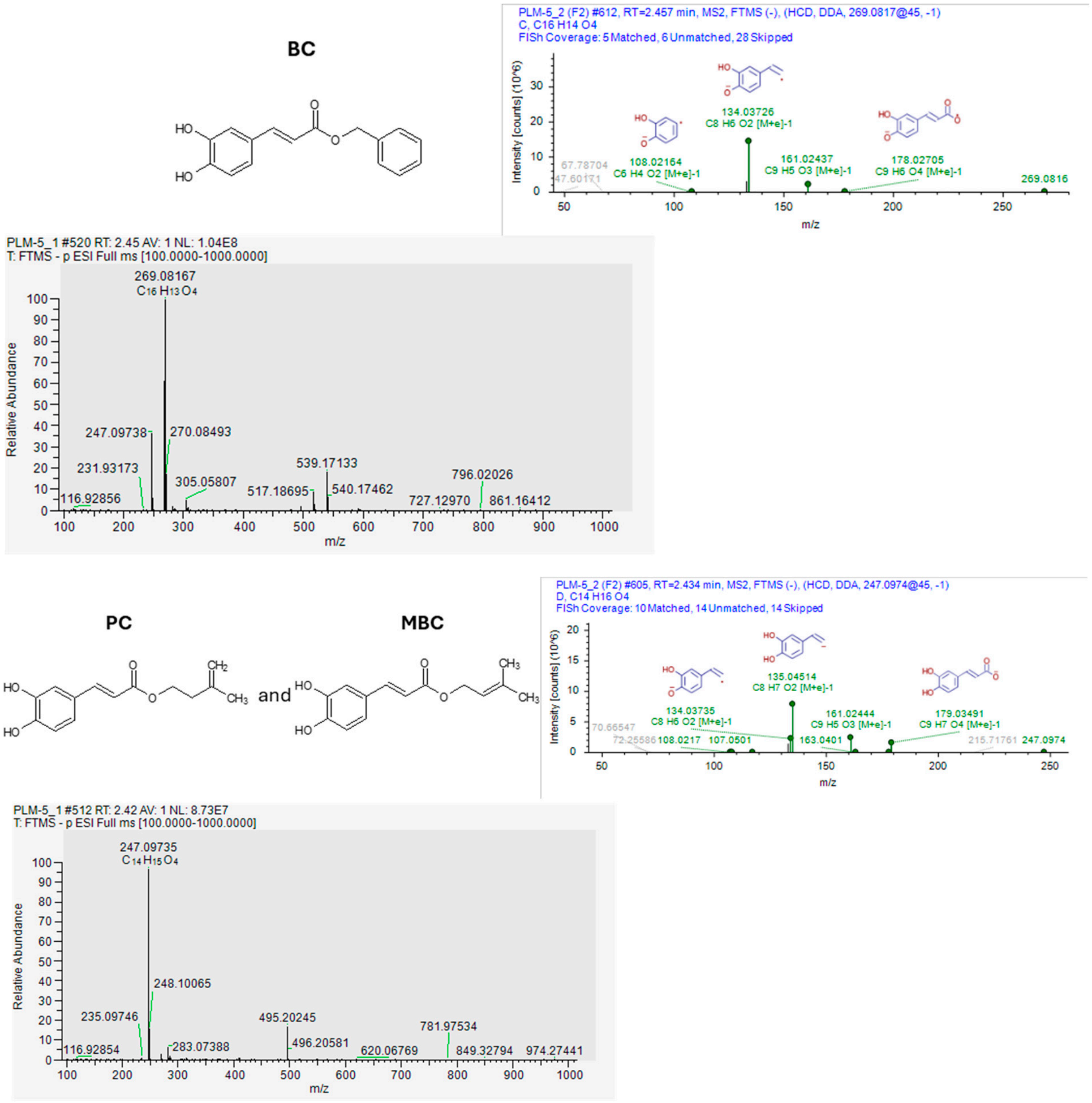


**Figure S2: LC-MS Analysis of Prop-A-2-4-2+3b fraction.**

Benzyl caffeate (BC), prenyl caffeate (PC), and (*E*) -3-methyl-3-butenyl caffeate (MBC).

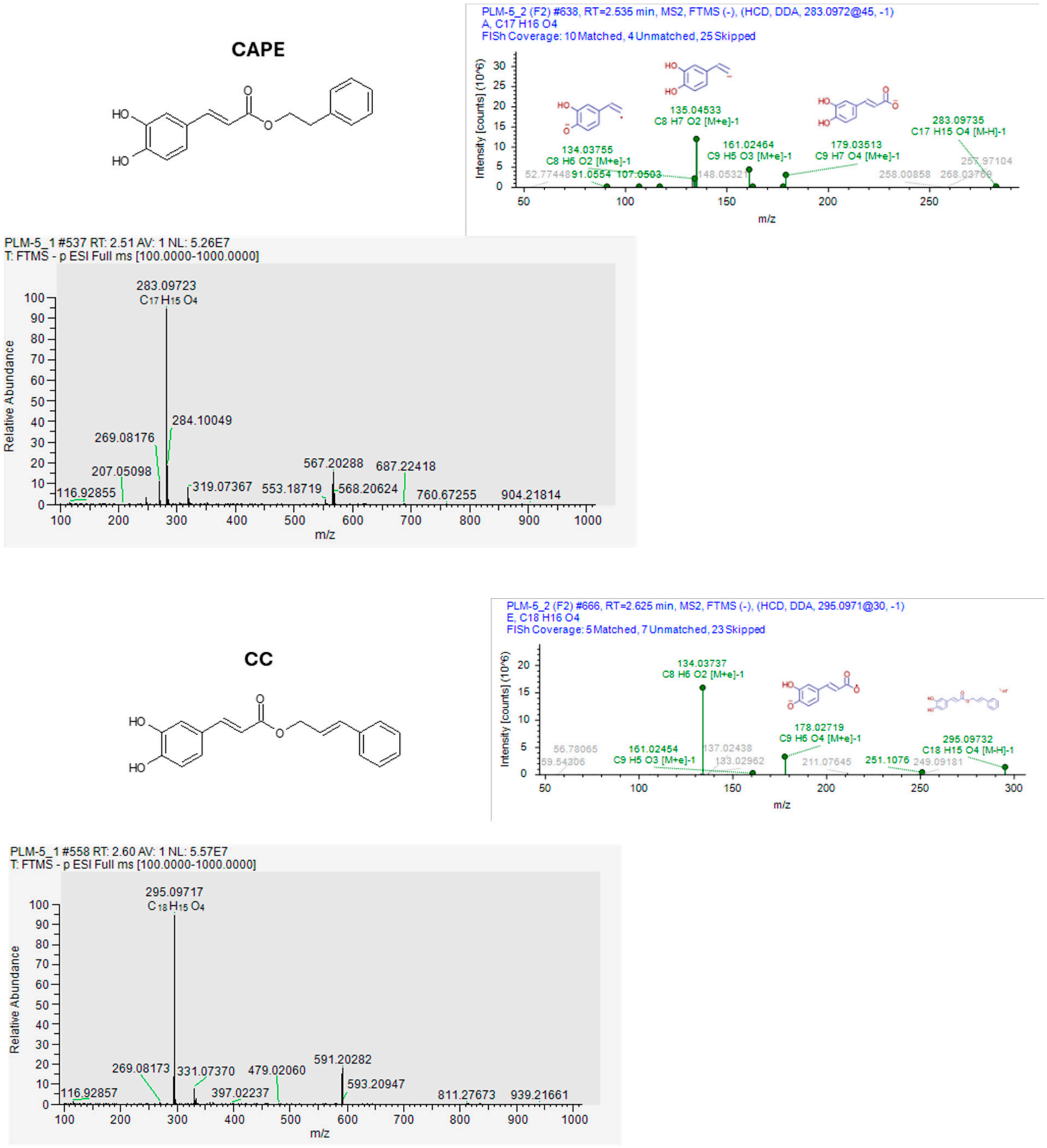


**Figure S3: LC-MS Analysis of Prop-A-2-4-2+3b fraction.**

Caffeic acid phenethyl ester (CAPE), 3-cinnamyl caffeate (CC).

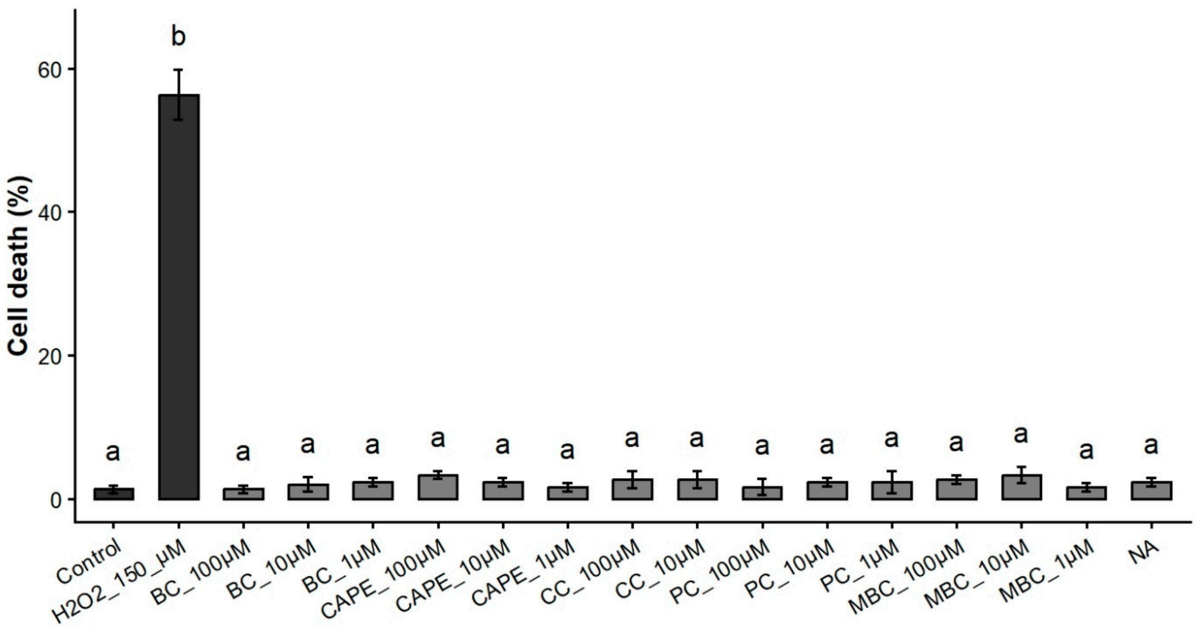


**Figure S4: Cytotoxicity of five caffeates standards.**

Cell death quantification (percentage of all cells) in human myoblasts incubated with all commercial standards at 1, 10 and 100 µM prior to incubation with $H_2O_2$. For each condition, three independent biological replicates were analyzed. Data are shown as mean ± SD (n = 3). Different letters indicate significant differences (one-way ANOVA followed by Tukey's HSD test, $p < 0.05$). Benzyl caffeate (BC), caffeic acid phenethyl ester (CAPE), 3-cinnamyl caffeate (CC), prenyl caffeate (PC), and (*E*)-3-methyl-3-butenyl caffeate (MBC).
